# A 10‐year analysis of MRI‐driven prostate cancer diagnosis and active surveillance: trends and implications

**DOI:** 10.1111/bju.16743

**Published:** 2025-04-14

**Authors:** Nikita Sushentsev, Rosita Comune, Kazim Ayberk Sinci, Oleg Blyuss, Iztok Caglič, Christof Kastner, Tristan Barrett

**Affiliations:** ^1^ Department of Radiology Addenbrooke's Hospital and University of Cambridge Cambridge UK; ^2^ Division of Radiology Università degli Studi della Campania Luigi Vanvitelli Naples Italy; ^3^ Department of Radiology Kanuni Sultan Suleyman Education and Research Hospital Istanbul Turkey; ^4^ Wolfson Institute of Population Health, Queen Mary University of London London UK; ^5^ Department of Urology Cambridge University Hospitals NHS Foundation Trust Cambridge UK; ^6^ Present address: Department of Paediatrics and Paediatric Infectious Diseases Institute of Child’s Health, Sechenov First Moscow State Medical University (Sechenov University) Moscow Russian Federation

**Abbreviations**
AS: active surveillance
GG: Grade Group
(cs)PCa: (clinically significant) prostate cancer
PI‐RADS: Prostate Imaging‐Reporting and Data System

Since the 2012 publication of the Prostate Imaging‐Reporting and Data System (PI‐RADS) recommendations [1], pre‐biopsy MRI has emerged as the first‐line diagnostic test in patients with suspected clinically localised prostate cancer (PCa). Initially endorsed by European and British guidelines in 2019 [2, 3] and subsequently reflected in the major North American guidelines [4, 5], this approach is supported by Level‐1 evidence showing MRI reduces unnecessary biopsies in patients without MRI‐identified suspicious lesions, while maintaining acceptable detection rates for clinically significant PCa (csPCa). These guidelines also recommend MRI for eligibility assessment and subsequent follow‐up of patients with PCa on active surveillance (AS), a management strategy with increasing global uptake [6].

In October 2014, our centre was among the first to implement dedicated MRI‐driven diagnostic and AS programmes. Here, we present their impact on csPCa detection and AS enrolment over a decade.

The local ethics committee approved this retrospective analysis of the local database, with consent taken from patients undergoing diagnostic procedures at our institution (Cambridge University Hospitals NHS Foundation Trust; Integrated Research Application System identifier [IRAS ID]: 313163 for pre‐biopsy MRI data, Cambridge University Hospitals [CUH]/18/3592 for AS data). Data were analysed from all patients undergoing pre‐biopsy prostate MRI during a 10‐year period (October 2014 to October 2024) (Fig. 1A). csPCa was defined as biopsy‐confirmed Grade Group (GG) ≥2 disease. Patients who commenced AS in our centre over the same period and had ≥12 months of follow‐up were also included (Fig. 1D). AS misclassification was recorded if a patient showed either radiological progression to ≥rT3 stage or histological progression to GG ≥3 disease within 12 months of AS enrolment. Considering the COVID‐19 disruption to imaging services, two observation periods were defined: pre‐pandemic (Years 1–5) and post‐pandemic (Years 7–10), with the latter also reflecting the impact of the 2019 national guidance recommending the omission of biopsies in patients without MRI‐visible disease [3]. A two‐sample *z*‐test for proportions was used to compare the relevant csPCa diagnosis and AS progression rates between these two independent periods.

**Fig. 1:**
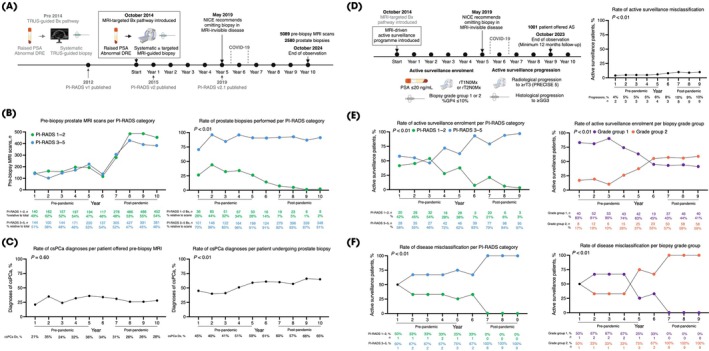
The 10‐year trends of MRI‐driven prostate cancer diagnosis and AS enrolment. **(A)** Timeline of the introduction of MRI‐driven PCa diagnostic pathway in our centre, resulting in 5089 pre‐biopsy (Bx) MRI scans and 2580 prostate biopsies performed over the 10‐year period. **(B)** Longitudinal plot presenting the number of positive (PI‐RADS 3–5) and negative (PI‐RADS 1–2) MRI scans (left), along with a plot presenting the percentage of patients with positive (PI‐RADS 3–5) and negative (PI‐RADS 1–2) MRI scans who underwent prostate biopsy in our centre over the observation period (right). **(C)** Longitudinal plots presenting the annual rates of csPCa diagnoses per patient offered pre‐biopsy MRI (left) and per patient undergoing prostate biopsy (right). **(D)** Timeline of the introduction of MRI‐driven AS pathway in our centre including the AS enrolment and progression criteria, resulting in the enrolment of 1001 patients on AS over the 9‐year period; a longitudinal plot is also given, presenting the proportion of patients enrolled on AS in our centre each year who experienced AS progression within the first 12 months of follow‐up. **(E)** Longitudinal plots presenting the rates of patients with positive (PI‐RADS 3–5) and negative (PI‐RADS 1–2) MRI scans (left) and biopsy‐confirmed GG1 and GG2 disease (right) enrolled on AS in our centre over the observation period. **(F)** Longitudinal plots presenting the annual rates of AS progression in patients with positive (PI‐RADS 3–5) and negative (PI‐RADS 1–2) MRI scans (left) and biopsy‐confirmed GG1 and GG2 disease (right). *P* values were derived using the two‐sided *z*‐test comparing the relevant proportions between the pre‐pandemic (Years 1–5) and post‐pandemic (Years 7–10) observation periods. NICE, National Institute for Health and Care Excellence.

Post‐pandemic, the considerable increase in pre‐biopsy MRI scans was balanced by the practice of limiting prostate biopsies almost exclusively to patients presenting with MRI‐visible (PI‐RADS 3–5) disease. As a result, the average proportion of patients with MRI‐invisible (PI‐RADS 1–2) disease who were offered prostate biopsy declined from 32% pre‐pandemic to 4% post‐pandemic (*P* < 0.01; Fig. 1B). Despite this shift, csPCa detection per pre‐biopsy MRI scan remained stable (*P* = 0.60), while the detection rate per biopsy increased significantly post‐pandemic (*P* < 0.01; Fig. 1C).

Similarly, a significantly higher proportion of patients on AS in the post‐pandemic period harboured MRI‐visible, GG2 disease compared to the pre‐pandemic period (average proportions: 90% vs 59%, 57% vs 22%, respectively; *P* < 0.01 for both; Fig. 1E). This shift was reflected in a significantly increased prevalence of these disease characteristics among misclassified patients showing early disease progression (*P* < 0.01 for both; Fig. 1F), consistent with the significantly increased rate of disease misclassification during the post‐pandemic period (*P* < 0.01; Fig. 1D).

This 10‐year analysis illustrates how MRI‐driven PCa diagnosis has reshaped the demographic and risk profile of newly diagnosed patients. Biopsy omission in patients without MRI‐visible disease significantly enhanced diagnostic efficiency, maintaining stable csPCa detection rates per MRI scan while increasing csPCa detection rates per biopsy. However, this approach led to increased enrolment of patients with MRI‐visible, GG2 disease on AS, contributing to a significant rise in short‐term progression to locally advanced (≥rT3) or intermediate‐unfavourable (GG ≥3) disease, indicating original disease misclassification. Although this affected no more than 10% of patients and the long‐term effect of such misclassification is unknown, this trend highlights the need for improving the risk‐stratification (rather than lesion detection) element of MRI‐driven PCa pathway. Doing so by integrating complementary approaches such as molecular imaging or tissue biomarkers to capture relevant tumour biology (e.g., cribriform morphology, *BRCA1*/*2* mutations) could preserve the benefits of biopsy omission while safeguarding patient outcomes.

## Funding Information

This research was supported by the UK National Institute for Health Research (NIHR) Cambridge Biomedical Research Centre (NIHR203312). The views expressed are those of the authors and not necessarily those of the NIHR or the Department of Health and Social Care. The authors also acknowledge support by Cancer Research UK Cambridge Centre, the Engineering and Physical Sciences Research Council Imaging Centre in Cambridge and Manchester, and the Cambridge Experimental Cancer Medicine Centre. Nikita Sushentsev acknowledges support from Emmanuel College, Cambridge. Oleg Blyuss acknowledges support from Barts Charity (G‐001522).

## Disclosure of Interests

The authors have nothing to disclose.
